# Tunable Fermi level and hedgehog spin texture in gapped graphene

**DOI:** 10.1038/ncomms8610

**Published:** 2015-07-27

**Authors:** A. Varykhalov, J. Sánchez-Barriga, D. Marchenko, P. Hlawenka, P. S. Mandal, O. Rader

**Affiliations:** 1 Helmholtz-Zentrum Berlin für Materialien und Energie, Elektronenspeicherring BESSY II, Albert-Einstein-Straße 15, 12489 Berlin, Germany; 2 Physikalische und Theoretische Chemie, Freie Universität Berlin, Takustraße 3, 14195 Berlin, Germany

## Abstract

Spin and pseudospin in graphene are known to interact under enhanced spin–orbit interaction giving rise to an in-plane Rashba spin texture. Here we show that Au-intercalated graphene on Fe(110) displays a large (∼230 meV) bandgap with out-of-plane hedgehog-type spin reorientation around the gapped Dirac point. We identify two causes responsible. First, a giant Rashba effect (∼70 meV splitting) away from the Dirac point and, second, the breaking of the six-fold graphene symmetry at the interface. This is demonstrated by a strong one-dimensional anisotropy of the graphene dispersion imposed by the two-fold-symmetric (110) substrate. Surprisingly, the graphene Fermi level is systematically tuned by the Au concentration and can be moved into the bandgap. We conclude that the out-of-plane spin texture is not only of fundamental interest but can be tuned at the Fermi level as a model for electrical gating of spin in a spintronic device.

## Abstract (web summary)

Potential electronic applications of graphene rely on controlling its spin-dependent properties. Here, the authors use spin-resolved photoemission spectroscopy to demonstrate how Au-intercalation produces gapped one-dimensional quasi-freestanding graphene on Fe(110) with tunable Fermi surface spin texture.

## Graphical abstract

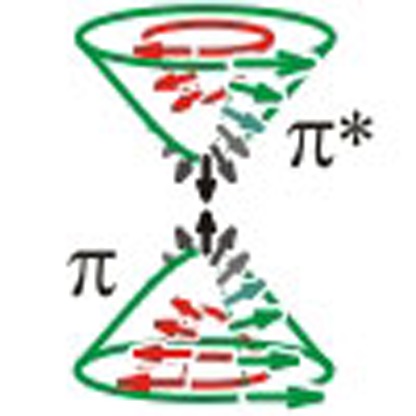

Graphene is a remarkably promising material for applications in electronics^1^. Its extremely compact Fermi surface consisting of only six points^2^ is very sensitive to external perturbations of the graphene lattice symmetry^3^,^4^ and to quantum-size effects arising from structural modulations^5^,^6^. This sensitivity in combination with the quasirelativistic character of the charge carriers^7^ has already lead to the realization of ultra-fast graphene-based transistors^8^,^9^ which can be improved even further if the control of the bandgap relative to the Fermi level is achieved through electrical gating of a graphene bilayer^10^. In the framework of a future spintronics, the question is how the use of these peculiar properties can be made. Graphene leads already to very large spin-relaxation lengths^11^ but, to become eligible as material in an active spintronics device, it requires also the ability to control the electron spin at the Fermi surface.

This requires three ingredients—lifting the spin degeneracy, enabling the spin currents and switching spin currents electrically. Ferromagnetic exchange is negligible in carbon and cannot create any substantial lifting of the spin degeneracy at the Fermi surface of graphene. Also the spin–orbit coupling is extremely weak (intrinsic band splitting ∼10^−5^ eV)^12^,^13^ but it can be enhanced by several orders of magnitude through contact of graphene to heavier elements and reach technologically relevant magnitudes^14^,^15^,^16^. In this way, we have earlier achieved for graphene on Ni(111) with an intercalated Au interlayer a Rashba-type spin–orbit splitting of the Dirac cone as large as ∼100 meV (ref. 15). In that system, the graphene is commensurate to the three-fold symmetric Ni(111) substrate. It is believed that for this reason the intercalation of Au affects neither the two-dimensionality nor the symmetry of the graphene resulting in a gapless Dirac cone^17^. This Dirac cone is quasi-freestanding meaning that the Fermi level is close to that of graphite. The degree of charge doping is independent of the amount of the intercalated Au^17^.

In the present work, we address the question of how the intercalation of heavy elements can be modified in a way that affects the symmetry of the graphene, its bandgap, its spin texture and its doping. In particular, we investigate by means of spin- and angle-resolved photoemission the intercalation of Au under graphene on Fe(110). We choose this system because it is known that graphene grows on Fe(110) with an anisotropic moiré pattern with giant (∼1.5 Å) height corrugation^18^. Indeed, we discover that the intercalation under such incommensurate graphene works very differently from that in the symmetric case of Ni(111) and endows graphene with a variety of novel properties, as we will show below—one-dimensional (1D) anisotropy, bandgap and together with a giant Rashba spin–orbit effect a spin texture turning out of the graphene plane.

In addition to rendering the graphene quasi-freestanding after intercalation of Au, the Au arrangement creates a significant bandgap (*E*_g_) at the Dirac point (*E*_g_∼230 meV). We attribute this behaviour to the reduced in-plane symmetry at the graphene–Au interface since we observe that the graphene also becomes 1D patterned as evidenced by a 1D arrangement of Dirac cone replicas in the band structure. Apparently this cannot occur unless the geometrical structure of the Au layer takes on the two-fold symmetry of the Fe(110) substrate. The bandgap, in turn, can be moved to the Fermi level since in this system the charge doping of the graphene changes with the concentration of the intercalated Au. Furthermore, we measured the photoelectron spin polarization directly. This measurement reveals a giant (Δ_S*O*_∼70 meV) spin–orbit splitting of the Dirac cone and a hedgehog-like out-of-plane reorientation of the spin within the gap, in line with recent theoretical predictions^19^. All in all, we demonstrate that the intercalation of nearly incommensurate graphene on Fe with different concentrations of Au effectively allows for the tuning of the size of the Fermi surface and its vectorial spin polarization in three dimensions.

## Results

### Electronic structure of intercalated graphene

At first, we have reproduced growth and characterization of graphene/Fe(110) (ref. 18) as a starting point for intercalation of the Au monolayer (ML). Graphene/Fe(110) has been shown to lead to a 1D corrugated structure due to the two-fold symmetry of the Fe(110) substrate. Low-energy electron diffraction (LEED) and scanning tunnelling microscopy demonstrate^18^ that this structure resembles an array of 1D stripes with a transversal periodicity of 38 Å, a giant height corrugation of 1.5 Å and a wave-like longitudinal pattern with periodicity of 17 Å along the stripes.

**Figure 1:**
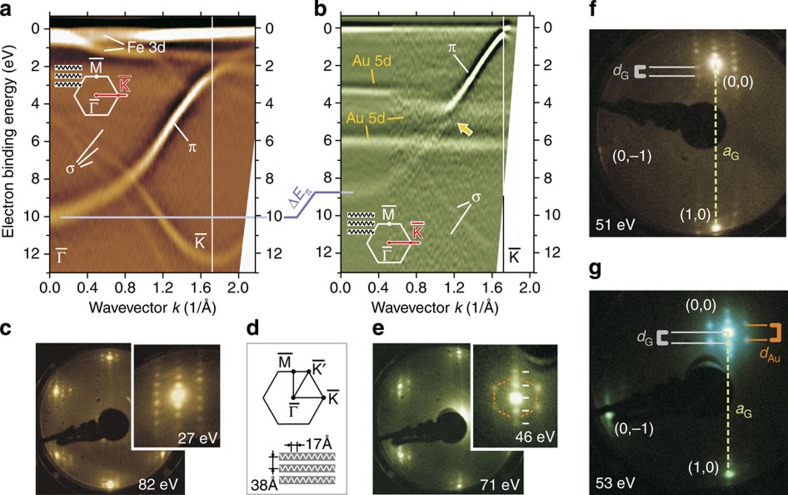
Modification of electronic and atomic structure of graphene on Fe(110) by intercalation of Au. (**a**) Band structure of graphene/Fe(110) before and (**b**) after intercalation of 1 ML of Au. Dispersions are measured along 
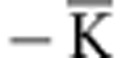
 direction of the graphene Brillouin zone (red line in the insets). After intercalation of Au, the *π* band undergoes an energy shift Δ*E*_*π*_≈1.2 eV (shown as magenta line) and hybridizes with Au 5*d* bands. Hybridization gap is pointed out in (**b**) with yellow arrow. (**c**,**e**) LEED patterns of graphene/Fe(110) (**c**) before and (**e**) after intercalation of Au reveal the enhanced 1D. Insets of **c** and **e** show zoom of moiré around (0,0) diffraction spot. (**d**) Sketch of the Brillouin zone and graphene stripes showing their orientation relative to LEED patterns displayed in (**c**,**e**,**f**,**g**). (**f**,**g**) In-depth analysis of LEED patterns of (**f**) bare and (**g**) Au-intercalated graphene/Fe(110). For the electron energies used (specified in the corners of panels), the main spots of zero (0,0) and first [(0,−1) and (1,0)] diffraction orders are observed together with the moiré-induced constellations. Real-space periodicity of graphene modulation corresponds to the distance *d*_*G*_ (in **e** denoted by dashes). The periodicity of superstructure of intercalated Au (denoted by an orange dashed hexagon in **e**) is given by *d*_Au_. Interrelations between *d*_*G*_, *d*_Au_ and *a*_*G*_ (which is the distance between main spots (0,0) and (1,0) and hence refers to graphene lattice constant) allow to determine the lateral periodicity of the 1D modulation of graphene occurring after intercalation of Au as 26 Å.

Similar to the cases of graphene on Ni(111) and on Co(0001) (ref. 20), graphene on Fe(110) interacts strongly with its substrate and has the bottom of the *π*-band shifted by ∼2 eV to higher binding energy as compared with free graphene (Fig. 1a). Intercalation of Au decouples the graphene from the Fe substrate. This is seen in Fig. 1b. The Au interlayer blocks the interaction between graphene and Fe and renders graphene quasi-freestanding meaning that the *π*-band undergoes a shift back toward lower binding energy (Δ*E*_*π*_∼1.2 eV). This apparent decoupling does not contradict hybridization with Au 5*d* states which is very pronounced as indicated in Fig. 1b by a yellow arrow. Peculiarities of this electronic hybridization are addressed more in details in Supplementary Note 1 and Supplementary Figure 1.

Also the moiré LEED patten of graphene/Fe changes remarkably after intercalation of Au. Figure 1e shows that after intercalation one new chain of moiré spots occurs (white dashes). This is different from the LEED pattern of bare graphene/Fe(110) (Fig. 1c), where multiple chains of spots present a 1D anisotropic structure with periodicities in both *x*- and *y* directions. This, in turn, indicates that intercalation of Au strongly enhances the 1D of the graphene structure. The role of the Au is revealed by the additional hexagon that appears around the graphene spot (orange line in Fig. 1e) and which we find not to be graphene derived but Au derived. Figures 1f,g show LEED patterns allowing for the simultaneous observation of moiré constellations and of principle spots of zeroth and first diffraction order from the graphene lattice and hence provide all information we need to determine the 1D periodicity of the new Au-intercalated graphene structure.

Figure 1g reveals that the 1D reciprocal-space periodicity *d*_G_ of the central graphene-derived chain of spots is smaller than the reciprocal-space periodicity *d*_Au_ corresponding to the Au superstructure. The distance between primary graphene spots [(0,0) and (1,0)] *a*_G_ is inversely proportional to the graphene lattice constant and allows to determine the moiré periodicity *d*_G_ before and after intercalation of Au. Analysis of a large set of LEED data reveals that after intercalation of Au *d*_G_ changes by a factor ∼0.7 which gives (27±2) Å for the real-space periodicity of the 1D modulation of the Au-intercalated graphene. The periodicity of the Au-derived pattern can correspondingly be extracted from *d*_Au_ and gives a value of (18±2) Å.

### 1D arrangement of band replica in photoemission

**Figure 2:**
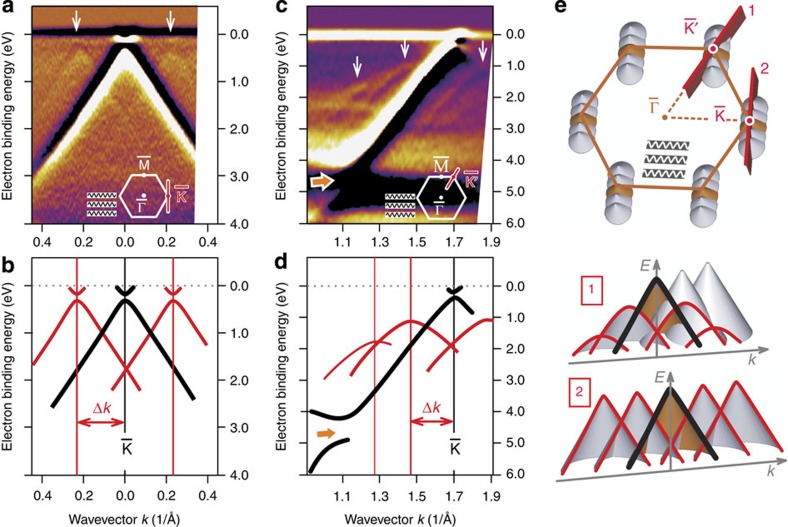
1D electronic structure of Au-intercalated graphene revealed by photoemission replicas of Dirac cones. (**a**,**b**) Band structure of graphene measured through the 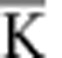-point and perpendicular to 
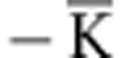
 directions of the Brillouin zone. This direction (shown in the inset to **a** by a red line) is also perpendicular to the 1D graphene. (**b**) Plot of the band structure extracted from **a**. In **a**,**b**, replicas of the Dirac cone (denoted by white arrows) are identical to the main cone at the 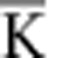-point and spaced equidistantly with periodicity Δ*k*=0.24 Å^−1^. (**c**,**d**) The band structure of Au-intercalated graphene sliced through the 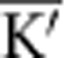-point and along the direction 
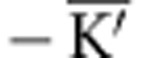
 (which is rotated by 60° to the direction of graphene stripes, as shown in the inset to **c**), gives replicas that occur miscut and extremely gapped. It is also seen that the replicas are not very periodic and Δ*k* is slightly reduced, as compared to (**a**,**b**). This effect is emphasized in **d**, which represents the band structure extracted from **c**. (**e**) ARPES data shown in **a**,**b** and **c**,**d** can only be explained if the arrangement of Dirac cones replica is 1D. Dirac cones appear in chains that are replicated perpendicular to the graphene stripes. Model sections of 1D replica chains along 
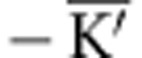
 (path 1) and perpendicular to 
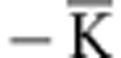
 (path 2; which correspond to ARPES data sets from **c** and **a**, respectively) reproduce the experimental data. Measurements were performed at a photon energy of 62 eV. ARPES intensity is enhanced by first derivative over energy. Yellow arrow in **c**,**d** denotes bandgap due to hybridization with Au.

The observation that the dimensionality of the Au-intercalated graphene is reduced from 2D to 1D is also confirmed by the observation of characteristic 1D replicas of the Dirac cone in photoemission band mapping. While no replicas are seen in the ARPES dispersion probed along the direction 
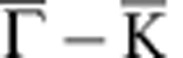
 of the surface Brillouin zone (Fig. 1b), clearly resolved replicas of the Dirac cone appear along the direction perpendicular to 
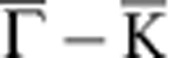
 (Fig. 2a,b). Note that the replica intensity is weak probably due to poor structural coherence of the graphene moiré on Au that is consistent with LEED. This anisotropy corresponds to a chain-like 1D arrangement of replicas around the 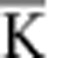-point as sketched in Fig. 2e (case 2 represents the dispersion cut from Fig. 2a). To cross-check this scenario, we have probed the band structure also through the 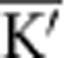-point along the 
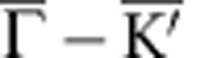
 direction (case 1 in Fig. 2e). This direction should provide an intact primary Dirac cone at 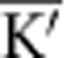 but all replicas must be miscut off centre and gapped. Exactly this is observed in the experiment (Fig. 2c,d). Note that the periodicity of the 1D superpotential extracted from the replica repetition in Fig. 2a,b as *D*=2*π*/Δ*k*=2*π*/(0.24 Å^−1^)=26 Å perfectly agrees with the periodicity of the 1D graphene moiré determined from the LEED pattern in Fig. 1g (27 Å). This behaviour is exactly what is expected from a 1D system. An additional information is that the Dirac cones are replicated along the direction transversal to the initial 1D structure of graphene on Fe(110) identified by Vinogradov *et al*.^18^ by its large corrugation in scanning tunnelling microscopy. These results show that it is the intercalation of Au that transforms the graphene into a 1D system both structurally and electronically and even mediates the periodicity of the 1D modulation. More details on the properties of band replicas can be found in Supplementary Note 2 and Supplementary Figure 2.

### Gapped Dirac cone and tunable charge doping

**Figure 3:**
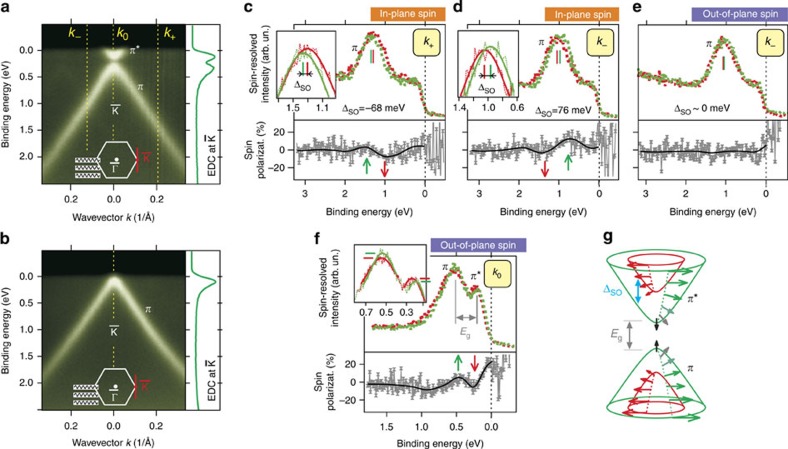
Observation of tunable doping and hedgehog spin texture in Au-intercalated graphene/Fe(110). (**a**,**b**) Dirac cones in graphene/Fe(110) intercalated with (**a**) 1 ML and (**b**) 2 ML of Au display a bandgap *E*_g_∼230 meV and different charge dopings. The effect is emphasized by intensity profiles (EDCs) sliced through the Dirac point. Increased concentration of Au makes graphene nearly charge neutral and moves the Fermi level into the gap. Red line in the Brillouin zone denotes the direction along which the dispersion was measured. (**c**–**e**) Spin-resolved photoemission of graphene intercalated with 1 ML of Au measured at positive *k*_+_ (**c**) and negative *k*_−_ (**d**,**e**) wave vectors relative to 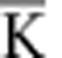. The panels display spin-resolved spectra together with the measured spin polarizations. Red and green colours and arrows in **c** and **d** denote opposite directions of spin circulation in the graphene plane. In **e**, the same colours denote out-of-plane spin components. For in-plane components the data in **c**,**d** reveals a giant spin–orbit splitting of the Dirac cone (Δ_S*O*_∼70 meV) which reverses its sign between *k*_−_ and *k*_+_. However, the out-of-plane spin splitting is zero (**e**). This clearly indicates Rashba physics. (**f**) Spin-resolved measurements of graphene intercalated with 1 ML Au taken within the bandgap at the Dirac point (wave vector *k*_0_). Here red and green denote spin components of opposite sign which are perpendicular to the graphene plane. Data reveal out-of-plane spin polarization of gap edges and evidence the formation of a hedgehog spin configuration (**g**).

The Au also mediates a concentration-dependent charge doping, which is known for graphene/Au/SiC^21^ but has not been seen on metal substrates so far. While for 1 ML of intercalated Au the graphene is *n*-doped and the Dirac cone is shifted toward higher binding energies (*E*_D_=*E*_F_−210 meV; Fig. 3a), for intercalation of nominally 2 ML it is charge neutral (*E*_D_=*E*_F_; Fig. 3b). Here *E*_D_ denotes the binding energy of Dirac point and *E*_F_ is the Fermi level.

In both cases of 1 ML and 2 ML of intercalated Au, the graphene possesses a bandgap at the Dirac point with size *E*_g_>200 meV (details of the bandgap determination through extrapolation scheme are given in the Supplementary Note 3 and Supplementary Figure 3). This is different from graphene/Au/Ni(111) which is gapless^17^. It is more similar to the case of graphene/Au/Ru(0001) that is also gapped^22^. We believe that the present bandgap at the Dirac point is related to a breaking of the sublattice symmetry of graphene due to the specific interface to the Au, which apparently is only two-fold symmetric. Our analysis of the ARPES data shows that after intercalation of 2 ML of Au the gap is aligned to *E*_F_, which means that already the concentration of Au effectively allows to tune the Fermi level of the graphene.

### Giant Rashba effect and hedgehog spin texture

The band structure of Au-intercalated graphene shown in Fig. 1b reveals electronic hybridization with Au. From our previous works^15^,^23^, we know that this hybridization enables a giant extrinsic Rashba effect on the graphene^24^,^25^, which leads to a peculiar band dispersion. We probe the Dirac cone of graphene/Fe(110) intercalated with 1 ML Au by means of spin-resolved photoemission and observe a giant spin–orbit splitting of these linear *π* bands of ∼70 meV. Spin-resolved spectra measured for negative (*k*_−_) and positive (*k*_+_) wave vectors (as referred to the 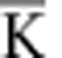-point; Fig. 3a) are reported in Fig. 3c–e. The spin polarization is observed only in the graphene plane and reverses its sign between *k*_−_ and *k*_+_, which is the fingerprint of the Rashba effect in graphene^24^,^25^,^26^.

When, instead, we probe the spin in the gapped Dirac point (wave vector *k*_0_), we observe an out-of-plane spin polarization, which reverses between upper and lower gapped Dirac cone (Fig. 3f). Together with the in-plane component, this leads to a hedgehog-type spin texture (Fig. 3g) similar to that expected for gapped topological insulators^27^. Further details of spin-resolved measurements can be found in Supplementary Notes 4–5 and Supplementary Figures 4–6.

## Discussion

For the gapped topological insulators, the hedgehog spin texture is caused by two ingredients. The first one is the in-plane spin texture due to spin-momentum locking and the second one is the breaking of time-reversal symmetry by a perpendicular exchange field. For the present case of graphene, again two ingredients are required for an out-of-plane spin reorientation, according to a recent theoretical investigation^19^. Rakyta *et al*.^19^ confirm that for ordinary Rashba-coupled graphene, the peculiar spin texture^24^,^25^,^26^ is expected to remain in the graphene plane, a result which is also in full agreement with the experiment^15^. If, however, the A−B sublattice symmetry of the graphene is simultaneously broken, Rakyta *et al*.^19^ predict the formation of a hedgehog-like spin texture at the gapped Dirac point. This is exactly what is observed in our present experiment.

The out-of-plane spin orientations of upper and lower Dirac cone are opposite to each other and the spin orientation is again reversed between 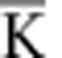 and 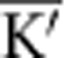. Being a valley effect^28^, this spin arrangement leads to the cancellation of Berry curvatures at 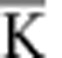 and 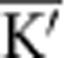. This is different from the cases of topological insulators with a single Dirac cone mentioned above^27^ or from the quantum anomalous Hall effect emerging in graphene when it is simultaneously subjected to strong spin–orbit and exchange interactions^29^. The quantum anomalous Hall effect in graphene leads to similar perpendicular spin components at the gapped Dirac points; however, Berry curvatures do not cancel at at 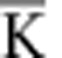 and 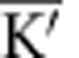 and additional topologically protected chiral edge states are expected when the graphene is in a ribbon geometry^29^.

**Figure 4:**
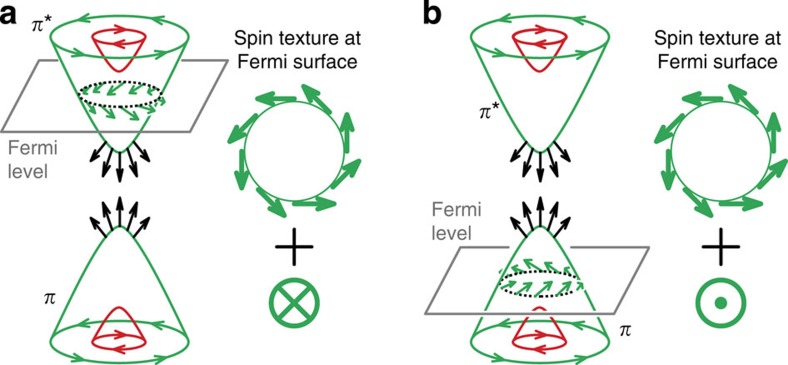
Effect of gating on the vectorial spin texture of graphene Fermi surface. Minor change of charge doping from (**a**) *n*-type to (**b**) *p*-type shifts Fermi level from the apex of upper gapped Dirac cone π* to the apex of lower cone *π* and switches the direction of out-of-plane spin while the in-plane spin texture remains unchanged. Red and green arrows sketch in-plane spins of Rashba split bands circulating in opposite directions. Black arrows denote hedgehog spins within the gap of the Dirac cone.

The hedgehog spin texture becomes the key to spin functionality in the context of Fermi level tuning which is shown here by varying the Au concentration (Fig. 3a,b). Aligning the bandgap to the Fermi level also tunes the vectorial spin texture of the graphene Fermi surface at 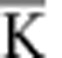 and 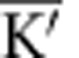 valleys, as illustrated in Fig. 4. This, in turn, is highly relevant for the emerging field of valleytronics dealing with electronic devices based on valley-dependent Berry phase effects^28^,^30^. In particular, spin-valley scattering in graphene with broken sublattice symmetry and non-equivalent magnetic moments of 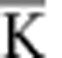 and 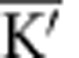 valleys may be used for the creation of highly effective spin separators (see Supplementary Note 6 and Supplementary Figure 7).

First of all, the valley Hall effect leads to a valley separation on application of an in-plane electric field^28^. The valley Hall effect^28^, however, does not take into account the electron spin. To explain how the spin texture observed in the present work combines with the valley Hall effect, we can refer to recent theoretical work on silicene^30^. The spin textures at 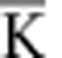 and 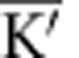 points predicted in silicene for an applied Zeeman field^30^ are in the current work already present in the ground state. The reversal of the spin texture is in the present case not achieved by reversing the Zeeman field but by tuning the Fermi level relative to the gapped Dirac cone so that the reversed spin texture at the upper or lower Dirac cone becomes effective at the Fermi level. The systematic doping effect of the Au discovered here is also a good demonstration for the feasibility of electrical gating of the hedgehog spin texture in a system where the present metallic Fe substrate is replaced by an insulator such as SiC.

In summary, we have investigated the intercalation of strongly corrugated moiré-type graphene on Fe(110) with Au. We demonstrated that the intercalation of Au decouples graphene from Fe and turns it quasi-freestanding. The intercalated Au forms a 1D superstructure at the interface and makes the quasi-freestanding graphene electronically 1D. Furthermore, the contact to Au introduces a bandgap of ∼230 meV in the Dirac cone. This bandgap can be moved to the Fermi level because the charge doping of graphene depends systematically on the Au concentration. Using spin-resolved photoemission we have shown that the Dirac cone of Au-intercalated graphene/Fe(110) exhibits a giant (Δ_S*O*_∼70 meV) Rashba-type spin–orbit splitting due to electronic hybridization with the Au. As a result of interplay between this giant Rashba effect and broken sublattice symmetry of graphene, the gapped Dirac cone reveals a hedgehog out-of-plane reorientation of electron spins. These findings indicate that the intercalation of strongly modulated graphene layers provides versatile possibilities for manipulation of electronic configurations and spin textures of graphene Fermi surfaces.

## Methods

### Sample growth

All sample preparations were done *in situ*. At first, a high-quality Fe(110) substrate was prepared as Fe overlayer on top of W(110). Fe was deposited on perfectly clean W(110) at low deposition rate (0.5 ML min^−1^) and subsequently annealed at 800 K for 5 min to improve the crystallinity of the Fe film. The amount of deposited Fe was verified by an oscillating quartz microbalance and varied between 20 and 40 MLs for different sample preparations. The W(110) substrate was initially cleaned by repeated cycles of annealing in oxygen (partial pressure of oxygen 1 × 10^−7^ mbar, temperature 1,500 K) followed by a short flash of the sample up to 2,300 K in ultra-high vacuum environment. Cleanliness of the W substrate was verified by the observation of surface-derived states in photoemission from 4*f* core level and from the valence band^31^.

Graphene on top of Fe(110) was synthesized by chemical vapour deposition of either ethylene or propylene^18^. The Fe sample was heated to 950–1,050 K in ultra-high vacuum. Then the hydrocarbon was rapidly admitted into the chamber at a partial pressure of 5 × 10^−6^ mbar for 5–10 min. The successful synthesis of graphene depends strongly on the partial hydrocarbon pressure and sample temperature. In the case of insufficient control over these parameters, an Fe surface carbide was forming.

For preparation of intercalated graphene/Fe(110), the Au was deposited on top of graphene and the sample was subsequently annealed at 800 K for 5 min. The amount of deposited Au was controlled by means of a quartz microbalance.

Extensive characterizations of the sample by ARPES and LEED at all stages of its preparation are reported in Supplementary Notes 7–8 and Supplementary Figures 8–9.

### Spin- and angle-resolved photoemission measurements

Photoemission experiments were performed at UE112-PGM1, UE112-PGM2, U125/2-SGM beamlines at synchrotron light source BESSY II using endstations ARPES 1^2^ and RGBL-II. Photon energies ranging from 30 to 100 eV and linear (s+p) polarization of light were used. The endstations were equipped with ARPES spectrometers Scienta R8000/R4000. The RGBL-II has a combined detector which comprises 2D channelplate for ARPES and Mott-type spin-detector for spin-resolved measurements. The spin detector is able to acquire both in-plane and out-of-plane components of photoelectron spins and can be operated simultaneously with ARPES acquisition of the band structure.

Overall resolutions of ARPES measurements were 5 meV (energy) and 0.3° (angular). Resolutions set for spin-resolved ARPES were 80 meV (energy) and 1.5° (angular). For positioning and angular orientation of samples in the ARPES experiment, a cryomanipulator operated either at *T*=25 K or at room temperature was used. Base pressure in all experimental chambers was below 4 × 10^−10^ mbar.

## Additional information

**How to cite this article:** Varykhalov, A. *et al*. Tunable Fermi level and hedgehog spin texture in gapped graphene. *Nat. Commun.* 6:7610 doi: 10.1038/ncomms8610 (2015).

## Supplementary Material

Supplementary Information Supplementary Figures 1-9, Supplementary Notes 1-8 and Supplementary References.
