# Supplementary material for: Tunable Fermi level and hedgehog spin texture in gapped graphene
Source: Nat Commun. 2015 Jul 27;6:7610. doi: 10.1038/ncomms8610 (PMC4525204; doi:10.1038/ncomms8610)
Supplement: Supplementary Information — Supplementary Figures 1-9, Supplementary Notes 1-8 and Supplementary References. [file ncomms8610-s1.pdf]

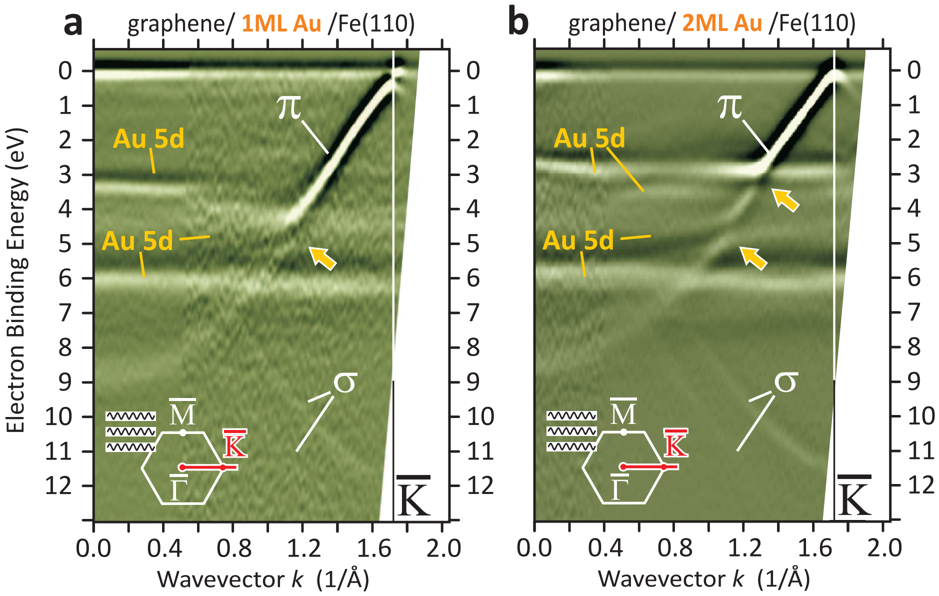

Supplementary Figure 1. Overall band structure of graphene/Fe(110) intercalated with (a) 1ML and (b) 2ML of Au measured by ARPES along the direction $\overline{\Gamma} - \overline{\mathrm{K}}$ of the surface Brillouin zone (SBZ). The intensity is enhanced through second derivative over energy. A modification of electronic hybridization between the π-band of graphene and 5d states of Au and the appearance of an additional hybridization gap at 3.2 eV for increased concentration of Au is seen (hybridization gaps are indicated by yellow arrows).

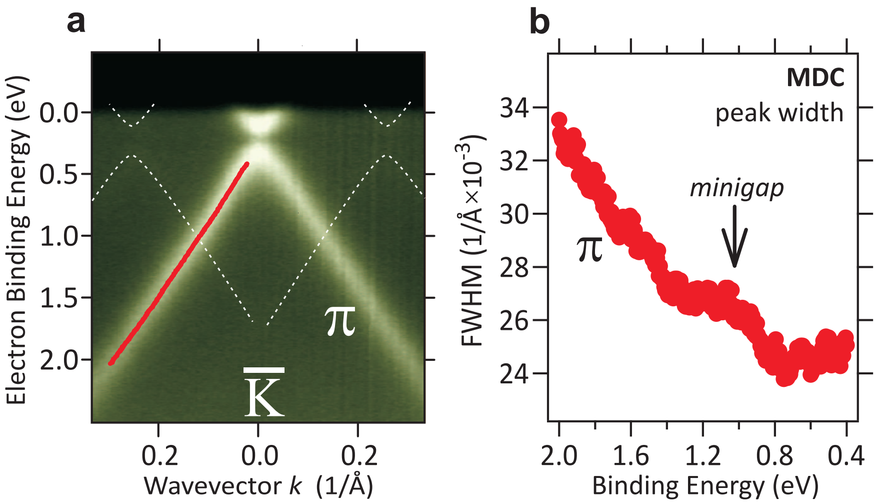

Supplementary Figure 2. Evidence for minigaps at the crossing of main Dirac cone with its 1D replica in graphene on Fe(110) intercalated with 1 ML of Au. (a) ARPES dispersion of Dirac cone (raw data). Weak band replica are marked with dashed lines. Positions of peaks extracted by fit from momentum-distribution-curve (MDC) profiles are denoted as red points. (b) Behaviour of MDC peak broadening with binding energy. A bump is observed at the energy corresponding to the crossing with replica (∼1 eV). This is assigned to an emerging minigap.

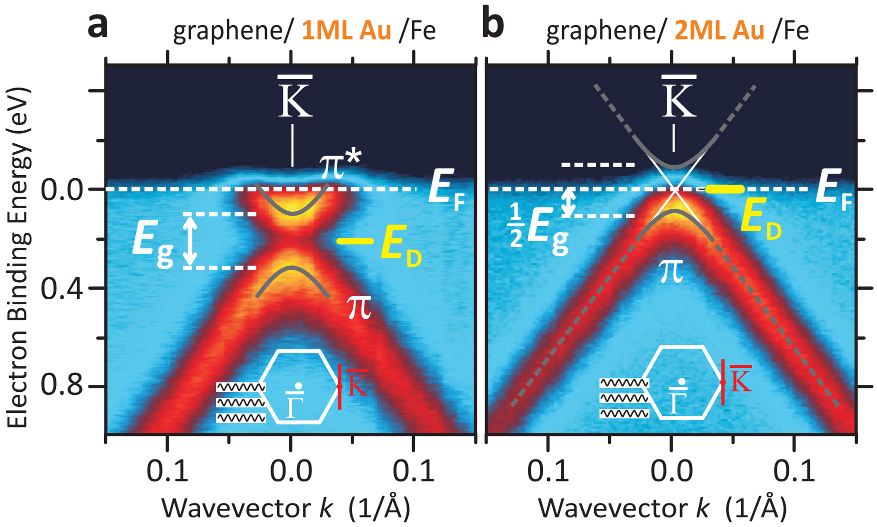

Supplementary Figure 3. Determination of band gap and charge doping in graphene/Fe(110) intercalated with (a) 1 ML (a) and (b) 2 ML of Au. (a) For 1 ML of Au charge doping is of n-type with Dirac point (E_{D}) located 210 meV below Fermi level (E_{F}). Determination of the band gap (E_{g}) is straightforward since both upper (π^*) and lower (π) cones are visible. Measured width of the gap is E_{g}=230 meV. (b) For 2 ML of Au an extrapolation scheme was used which reveals charge neutrality of graphene and $E_{\mathrm{g}}\sim$205 meV. Sketches in the insets depict the direction within the Brillouin zone along which the dispersions were acquired (red lines).

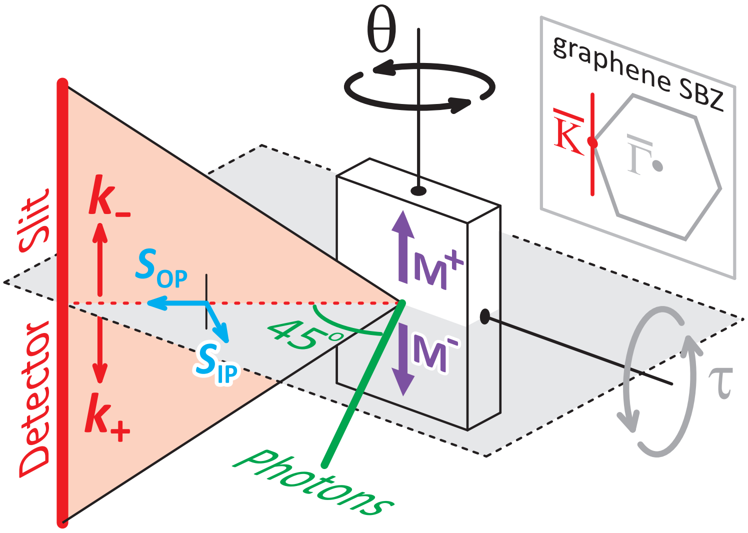

Supplementary Figure 4. Geometry of the spin resolved photoemission experiment. Detector slit (slice of the band structure dispersion) is vertical. Detection plane of photoelectrons along which the dispersion with wavevectors k is acquired is shown in light red. Incoming beam of photons is in horizontal plane (gray) and is denoted as green line. Spin detector acquires in-plane and out-of-plane spin components denoted as S_{IP} and S_{OP}, respectively. Easy axis of magnetization of Fe film (M^+ or M^-) was aligned to vertical direction. This ensures that any arbitrary rotation of the sample around the polar axis (θ) does not produce projection of spins from 3d states of Fe onto S_{OP} axis. Corresponding orientation of graphene surface Brillouin zone (SBZ) is shown in the back.

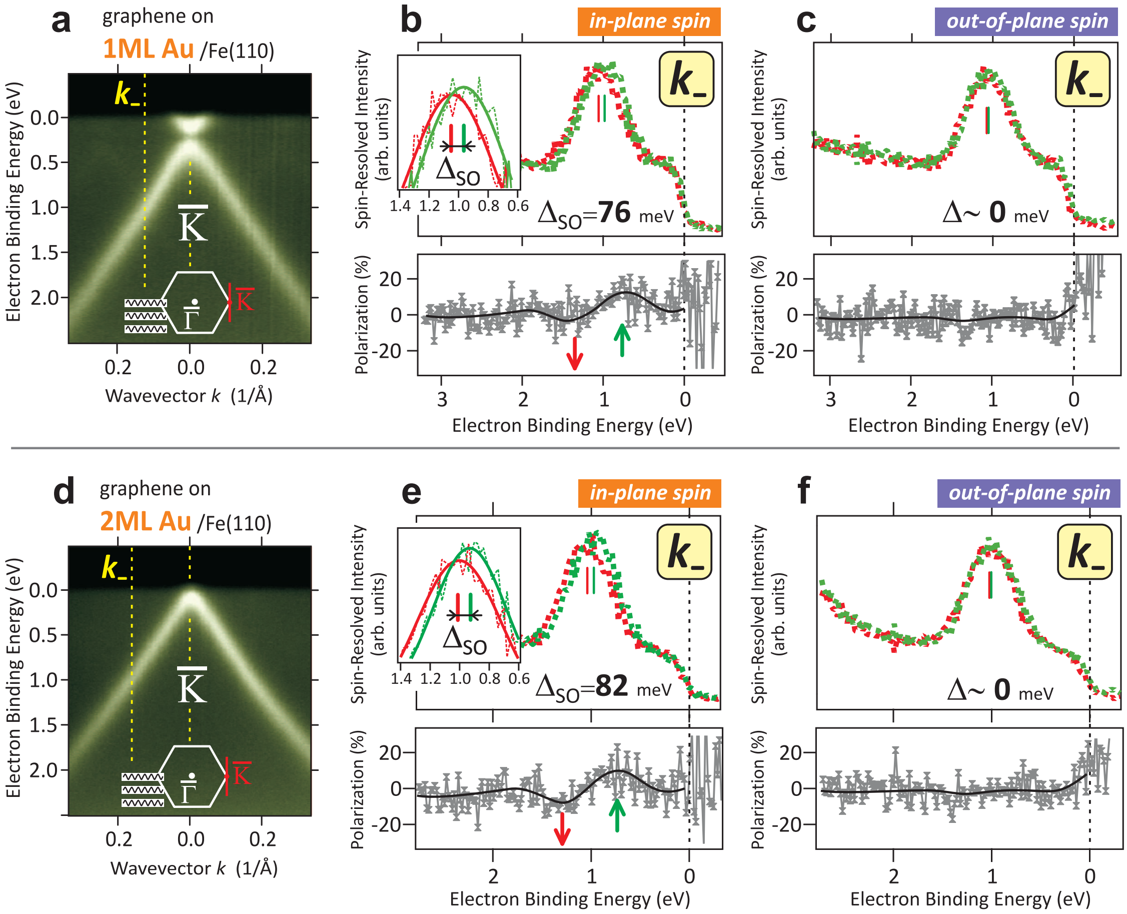

Supplementary Figure 5. Spin-orbit splitting of Dirac cone in graphene/Fe(110) intercalated with (a-c) 1 ML and (d-f) 2 ML of Au measured away from $\overline{\mathrm{K}}$-point. (a) ARPES dispersion (raw data) of graphene intercalated with 1ML of Au. Line k_- denotes wave vector k for which the spin resolved spectra displayed in (b) and (c) were measured. (b) Energy-distribution-curve (EDC) spectrum resolved for in-plane spin component S_{IP} at k_-. (c) EDC spectrum resolved for out-of-plane spin component S_{OP} at k_- acquired simultaneously with spectrum from (b). Measured spin polarizations are also shown. Red and green arrows and points denote spin components of opposite sign. (d,e,f) Photoemission measurements equivalent to (a,b,c) but performed for graphene intercalated with 2 ML of Au. It is seen that independently of the concentration of Au the in-plane spin polarization reveals giant spin-orbit splitting Δ_{SO} of the order of 70 meV (e). At the same time the out-of-plane spin polarization is zero (f). This picture where k is probed away from the Dirac point is consistent with the scenario of the Rashba effect for graphene.

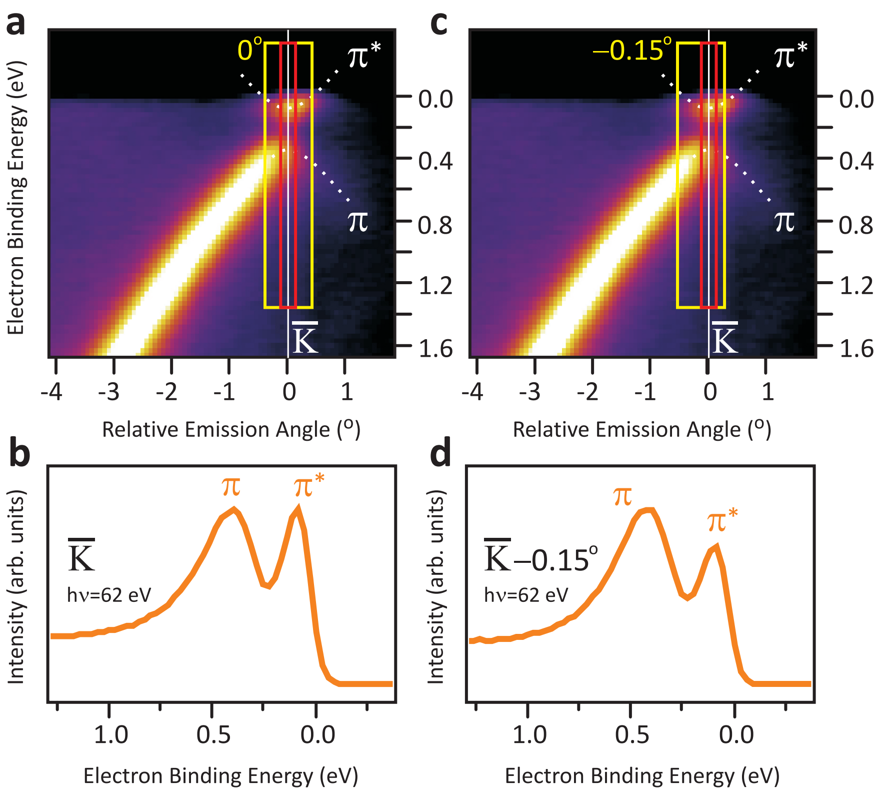

Supplementary Figure 6. Estimation of experimental errors due to sample misalignment of the spin-resolved measurements at $\overline{\mathrm{K}}$-point of graphene surface Brillouin zone (SBZ). (a) ARPES dispersion of Dirac cone sampled along $\overline{\Gamma}-\overline{\mathrm{K}}$ of graphene SBZ. Anisotropic intensity of π and π^* bands due to Brillouin zone effects is seen. Yellow frame denotes acceptance frame of spin analyser positioned precisely at $\overline{\mathrm{K}}$-point. Red frame denotes angular localization of spin hedgehogs around $\overline{\mathrm{K}}$. (b) Spin-resolved EDC spectrum simulated by integration of ARPES intensity over yellow frame reveals equal intensity of π and π^* peaks. (c,d) Same as (a,b) but yellow frame of spectrometer is misaligned by 0.12° toward 1st SBZ. Intensity disbalance between π and π^* is identical to that seen in spin-resolved spectrum in Figure 3(f) (in the article), but red frame of spin hedgehog remains perfectly inside of yellow frame of analyser and therefore is fully acquired. Photoemission measurements were performed with photon energy of 62 eV.

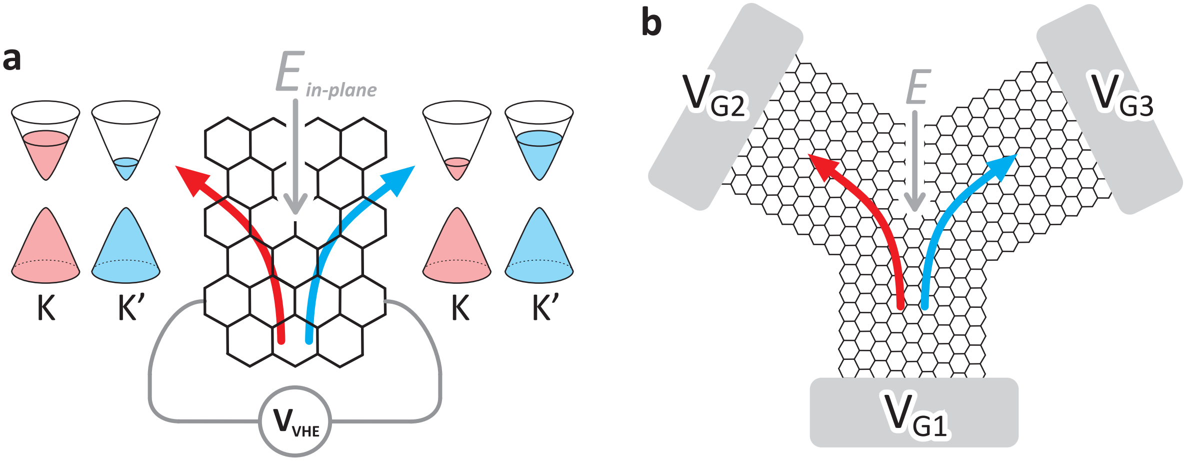

Supplementary Figure 7. Relevance of spin hedgehogs in graphene to spintronics. (a) Valley Hall effect (VHE) emerging in graphene with inequivalent orbital magnetic moments of $\overline{\mathrm{K}}$ and $\overline{\mathrm{K}'}$ valleys. When the electric field $E_{\mathrm{in-plane}}$ is applied in the graphene plane and collinear to the current direction, deflection of the charge carriers toward the edge of the stripe occurs due to valley-polarized scattering induced by a Berry phase effect. This causes different population of Dirac cones at $\overline{\mathrm{K}}$ and $\overline{\mathrm{K}'}$ valleys at opposite edges of the graphene stripe. This idea was firstly formulated in Ref. [20] but for valley associated pseudo spin and not for real spin. (b) Scheme of Y-shaped spin separator utilizing the effect of spin-valley scattering for spin filtering of electric currents. The spin separator is attached to three conducting gates/leads. Gates 2 and 3 (collectors) have equal potentials V_{G2} and V_{G3}. Voltage at gate 1 (emitter) V_{G1} is different. The difference between V_{G1} and $V_{\mathrm{G2,G3}}$ drives electric current through Y-shaped graphene flake, but, at the same time, creates an in-plane electric field which activates spin-valley scattering. As a result, charge carriers with one spin are deflected toward gate 2 (red arrow) and charge carriers with the opposite spin toward gate 3 (blue arrow). Principle of valleytronic device utilizing spin-valley scattering was proposed in Ref. [21] but for using Zeeman effect for spin-polarization of $\overline{\mathrm{K}}$ and $\overline{\mathrm{K}'}$ valleys and not spin hedgehogs (which in present study are already available in ground state without application of external field).

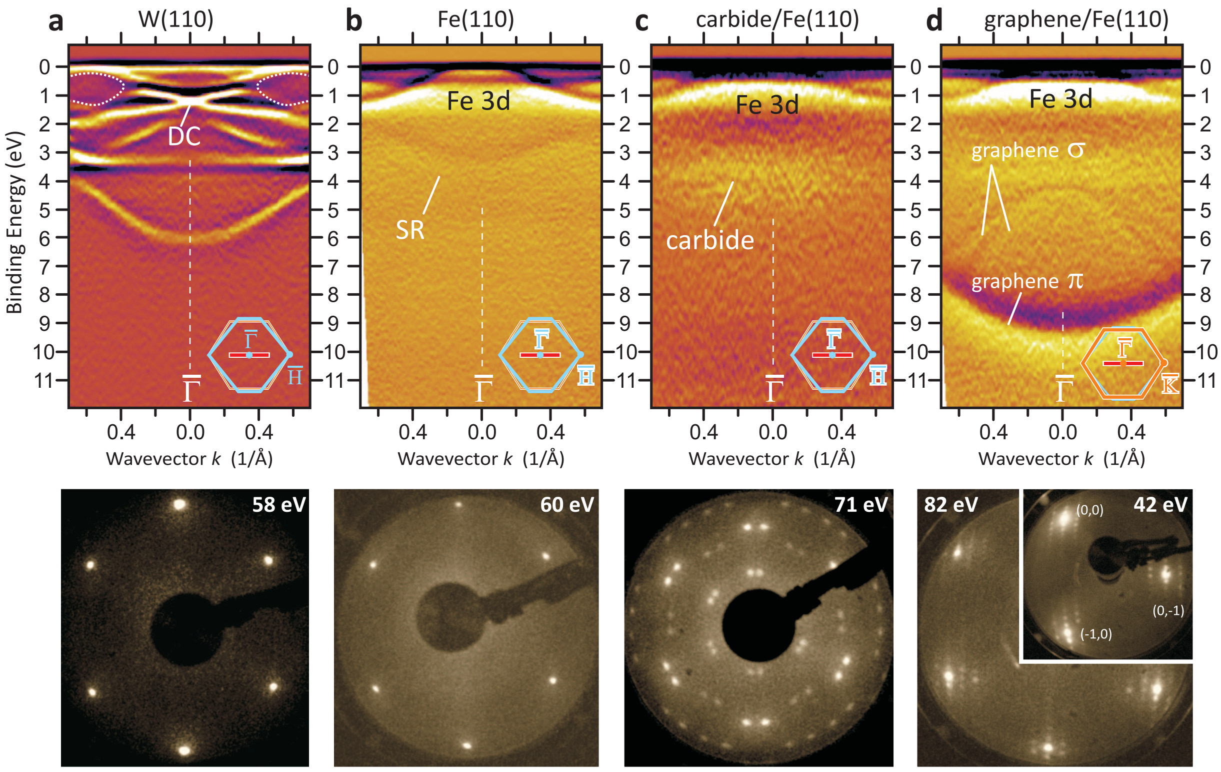

Supplementary Figure 8. Characterization of various stages of sample preparation by angle-resolved photoemission (upper panels) and by low-energy electron diffraction (lower panels). (a) Clean W(110). Perfect cleanliness of W(110) is confirmed by the observation of renowned Dirac-cone-like surface state (DC) at 1.5 eV. (b) Fe(110) prepared as 20-ML-thick Fe film on top of W(110). High structural quality of Fe(110) is evidenced by the presence of surface resonance (SR) and by sharp (1×1) pattern in LEED. (c) Fe(110) surface carbide produced as a result of chemical vapour deposition (CVD) of propylene at too low partial pressure. Carbidic band at 3.6 eV is broad and dispersionless. Its LEED pattern is complex. (d) High quality graphene/Fe(110) with characteristic moiré pattern seen in LEED and pronounced dispersions of σ and π graphene states in the valence band. ARPES intensity in (a) is enhanced through second and in (b-d) through first derivative over energy. Insets of panels with ARPES data depict Brillouin zones of (110)-face of W and Fe (blue) and graphene (orange). Directions along which the dispersions were measured are shown in the insets by red line. A photon energy hν=62 eV was used. Electron energies used for LEED measurements are specified in the corners of images.

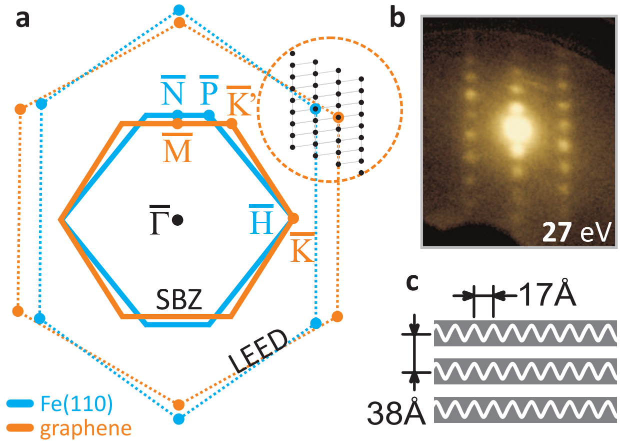

Supplementary Figure 9. Origin of moiré pattern in graphene/Fe(110). (a) graphical superposition of LEED patterns from Fe(110) [blue dashed line] and from graphene [orange dashed line] emphasizes different lattices. The (7×17) moiré pattern occurs as a replication of principle diffraction spots with periodicities determined by lattice differences (see plot in orange circle). Surface Brillouin zones (SBZ) corresponding to Fe(110) and graphene are plotted with blue and orange thick solid lines, respectively. (b) Zoom of the LEED image with (7×17) moiré pattern around principle spot. (c) Sketch of graphene stripes with longitudinal wave-like pattern in orientational correspondence to the LEED image.

Supplementary Note 1

Electronic hybridization between graphene and Au

Supplementary Figure 1 displays the overall band structure of graphene/Fe(110) intercalated with 1ML [Supplementary Figure 1(a)] and 2ML of Au [Supplementary Figure 1(b)] sampled along the direction $\overline{\Gamma} - \overline{\mathrm{K}}$ of the surface Brillouin zone (SBZ). After increasing the concentration of Au the π-band moves toward lower binding energies. At the $\overline{\Gamma}$-point it shifts from 8.9 eV to 8.6 eV. Also at $\overline{\mathrm{K}}$ the upper Dirac cone moves upward above the Fermi level. One can also see that the hybridization between the π-band of graphene and 5d states of Au changes with increasing concentration of Au.

While for 1ML of intercalated Au only one hybridization gap at $\sim$ 4.5 eV is seen [yellow arrow in Supplementary Figure 1(a)] after increasing the amount up to 2ML a second hybridization gap at $\sim$ 3.2 eV appears [yellow arrows in Supplementary Figure 1(b)]. Such behaviour is a hallmark for the structural transition of Au the interlayer under graphene towards a superstructure with a new periodicity. Similar effects were observed for Au-intercalated graphene on Ni(111) where the appearance of new hybridization gaps was related to a structural change of the Au interlayer from (9×9) to (8×8) superstructure [5]. Unique with graphene/Fe(110) is thus a structural transition of the Au interlayer toward a superstructure with higher density which also changes the charge doping of graphene.

Supplementary Note 2

Dirac cone replicas and minigaps

Figure 2 of the main article shows that ARPES dispersions of graphene/Fe(110) intercalated with Au reveal 1D replicas of the Dirac cone. These replicas are repeated along the direction perpendicular to the graphene stripes. For other moiré-type graphene systems [e.g. graphene/Ir(111)] it is known that energy gaps occur as minigaps at the crossings of replicas with the main Dirac cone [8–11]. The width of the minigaps is determined by the amplitude of the modulating lateral superpotential [9, 12].

Hence we have inspected the Dirac cone in graphene/Fe(110) intercalated with 1ML of Au for the presence of minigaps. Supplementary Figure 2(a) shows ARPES dispersion measured along the direction of the surface Brillouin zone which is perpendicular to $\overline{\Gamma} - \overline{\mathrm{K}}$ (same dispersion as displayed in Figure 2(a) of the main article but here shown as raw data). Replica bands are denoted with white dashed lines. One can see that they cross with the primary Dirac cone at a binding energy of ~ 1 eV. Due to the weakness of the replica (related to poor coherence of graphene to the intercalated Au interlayer) the minigaps can unlikely be detected by direct analysis of the intensity. Therefore we have analysed the broadening of the photoemission peak of the π-band in terms of momentum distribution curves scans along k. This broadening physically represents the imaginary part of the self-energy Σ.

Red dots superimposed on the dispersion in Supplementary Figure 2(a) denote fitted peak positions. Peak widths extracted from momentum distribution curves (MDC) are plotted in Supplementary Figure 2(b). Instead of a monotonic decay expected for the self-energy Σ of freestanding graphene (or graphite) [13] a pronounced bump is observed at ~ 1 eV binding energy. This is exactly the energy where the replica crosses with the primary Dirac cone. We attribute this broadening of the MDC peak to an emerging minigap. This makes the ARPES results for graphene/Au/Fe(110) consistent with the results from the literature for graphene/Ir(111) and permits to ascribe the 1D replication of Dirac cone to the ground state of the system [8–10] rather than diffraction of photoelectrons in the final state of photoemission.

Supplementary Note 3

Determination of band gap and doping in the Dirac cone

Supplementary Figure 3 shows ARPES spectra of Dirac cones of graphene/Fe(110) intercalated with 1ML [Supplementary Figure 3(a)] and 2ML of Au [Supplementary Figure 3(b)] on an enhanced scale. Dispersions are measured along the direction perpendicular to $\overline{\Gamma} - \overline{\mathrm{K}}$ line of the surface Brillouin zone. This, in contrast to the measurements along $\overline{\Gamma} - \overline{\mathrm{K}}$ [Supplementary Figure 1], allows for the observation of both sides of the Dirac cone due to diffraction of photoelectrons [6, 7]. We took special care to ensure that dispersions shown in Supplementary Figure 3 cut the Dirac cones precisely through $\overline{\mathrm{K}}$. In particular, full photoemission mappings of Dirac cones $I(E, k_{\mathrm{x}}, k_{\mathrm{y}})$ were performed and emission angles corresponding to $\overline{\mathrm{K}}$ were carefully determined.

The determination of the band gap E_{g} and binding energy of the Dirac point E_{D} is unproblematic for graphene intercalated with 1ML of Au [Supplementary Figure 3(a)]. Indeed, due to n-type doping both cones of lower π and upper π^* bands are observable and the center (Dirac energy E_{D}) as well as the width of the gap E_{g} can be read directly from the ARPES dispersion. For 1ML Au we acquire E_{D}=E_{F}−210 meV and E_{g}=230 meV.

The case of 2ML of intercalated Au is more complicated. Supplementary Figure 3(b) shows that the lower Dirac cone demonstrates a curvature just below E_{F} (lower edge of the gap) but the upper cone of π^* band has moved above the Fermi level and neither width of the gap between π and π^* nor middle of the gap can be read directly from the ARPES dispersion. In this case we apply an extrapolation scheme assuming that upper and lower Dirac cones are mirror symmetric. Such scheme provides an estimate for the minimal value of E_{g}. As shown in Supplementary Figure 3(b) the linear dispersion of the π-band is extrapolated by straight lines and the crossing point between them is taken as Dirac energy E_{D}. The width of the gap E_{g} is determined as twice the difference between E_{D} and the lower edge of the gap. In this way we obtain E_{D}=E_{F}(±10 meV) meaning that graphene is charge neutral and E_{g}=205 meV.

Supplementary Note 4

Characterization of Au-intercalated graphene by spin-resolved ARPES

Supplementary Figure 1 reveals pronounced electronic hybridization between the π-band of graphene and 5d states of Au. Indeed, we have earlier identified such hybridization as a precondition for a Rashba effect in graphene [14]. Hence we have extensively investigated Au-intercalated graphene on Fe(110) by means of spin-resolved photoemission.

The geometry of the spin resolved experiment is sketched in Supplementary Figure 4. Detection plane of the ARPES spectrometer (direction of ARPES slice) is vertical and the dispersion is acquired for electrons propagating along the slit with wavevectors k_- and k_+. The slit is denoted by a thick solid red line. The angle between incoming photons and detection plane of the spectrometer is 45°.

The Mott-type spin detector is installed in a configuration allowing for detection of one spin component S_{IP} which is perpendicular to the analyser slit and of another one S_{OP} which is parallel to the spectrometer axis (dashed red line). In the typical situation when spectra are measured close to normal emission, S_{IP} represents the spin component in the plane of sample surface and perpendicular to the momentum k of the photoelectron. S_{OP} is the projection of the spins on the axis perpendicular to the sample surface. Hence the notations in-plane spin and out-of-plane spin are used throughout the paper in order to denote S_{IP} and S_{OP} components of spin. For spin-resolved measurements of the Dirac cone the sample was oriented in such a way that direction $\overline{\Gamma}-\overline{\mathrm{K}}$ of the surface Brillouin zone was horizontal and the detector slice was brought precisely to $\overline{\mathrm{K}}$ by rotation of polar angle θ. The spin-orbit splitting was then tested for wavevectors k_- and k_+ by tilting of the sample (change of angle τ by a few degrees up and down).

Supplementary Figure 5 reports spin-resolved measurements of the Dirac cone in graphene intercalated with 1ML [Supplementary Figures 5(a-c)] and 2ML of Au [Supplementary Figures 5(d-f)]. The data was acquired for wavevector k_- slightly away from $\overline{\mathrm{K}}$. In both cases a giant band splitting of $\sim$70 meV is observed for in-plane spins (S_{IP}) [Supplementary Figures 5(b,e)], but the measured out-of-plane spin polarization S_{OP} is zero [Supplementary Figures 5(c,f)]. Such behaviour fully complies with the scenario of the Rashba effect in graphene [15, 16]. We should emphasize that the measurements of in-plane and out-of-plane spin components are feasible despite certain polar rotation of the sample (θ) toward $\overline{\mathrm{K}}$. There is

only minor projection of in-plane spins onto the S_{OP} axis while measuring an out-of-plane signal because the rotation of θ toward $\overline{\text{K}}$ is small ($\theta \sim 24°$ at photon energy hν ~60 eV) and additionally reduced by non-zero tilt τ. The resulting magnitude of out-of-plane projection is less than $\frac{1}{3}$ of the in-plane component and nearly undetectable.

Particular care was taken to ensure the correct observation of the hedgehog-type out-of-plane spin texture at $\overline{\text{K}}$. Although projection of Rashba-type (in-plane) spin polarization onto S_{OP} axis is negligible, one may naively argue that the spin polarization of exchange-split 3d bands in the underlying Fe film may contribute. In order to exclude such possibility we have oriented the easy axis of Fe magnetization (for thin Fe layers on W(110) it is aligned to [1$\bar{1}$0] of Fe(110) in the plane of the Fe film [17]) along the axis of polar rotation θ and performed measurements for non-magnetized samples (this means co-existence of domains with opposite magnetizations M^+ and M^-). As one sees from Supplementary Figure 4 this experimental geometry guarantees that independently on rotation of θ magnetizations M^+ and M^- (and spins of electrons from Fe) remain perpendicular to S_{OP} axis and cannot contribute to the out-of-plane spin signal.

Furthermore, ARPES [Supplementary Figures 5(a,d)] clearly shows that the intercalated Au interlayer brings Fe out of range of the probing depth of photoemission and completely suppresses photoemission from Fe 3d reducing its potential contribution to negligible values. Absence of contributions from Fe is ultimately evidenced by the spin-resolved data in Supplementary Figures 5(c,f). Spectra do not reveal any Fe-related out-of-plane spin polarization for small tilt of the sample ($\tau \sim \pm 2°$). Hence there can be no contributions from Fe for τ=0° ($\overline{\text{K}}$-point) as well.

Finally, we want to emphasize that the measured hedgehog-type spin textures originate from the outer band of spin-orbit spilt Dirac cone, which is sketched in Figure 3(g) of the article by green lines. The out-of-plane spin reorientation within the gap of inner Dirac cone [red lines in Figure 3(g)] according to estimations given in Ref. [18] would be very small and under detection limit. It can not contribute in spin-resolved measurements in any substantial way.

Supplementary Note 5

Precision of sample alignment

We would like to comment on the momentum resolution of the spectrometer and on the accuracy of sample orientation, and show that small experimental errors are negligible for the correct interpretation of our spin- and angle-resolved photoemission measurements. Spin-resolved spectra revealing out-of-plane spin polarization (spin hedgehog) in the gap of the Dirac cone [Figure 3(f) in main article] display slightly different intensities of lower (π) and upper (π^*) bands at the gap edges. However, precisely at $\overline{\mathrm{K}}$, intensities of upper and lower cones have to be equal, as ARPES data in Figure 3(a) in the article shows. This suggests that the sample had slight angular misalignment in the spin-resolved measurement. This small misalignment originates from the transfer lens setup (aperture positioning) of our state-of the-art spectrometer which allows for simultaneous acquisition of ARPES dispersions and spin-resolved EDCs without changing the sample position. This is an important feature of the spectrometer and the small misalignment is, hence, principally unavoidable. Our analysis below shows that the resulting experimental mistake is negligible and has no effect on the results obtained.

The error introduced by the transfer lens is easily estimated by the angular dependence of the ARPES signal. Relatively large differences of the intensities of upper and lower Dirac cone in the gap result already from a minor angular misalignment of the the sample. The reason for this is the distribution of photoelectron intensity in the Dirac cones, which is extremely anisotropic due to a Brillouin zone effect [6]. Due to this effect, the intensity of the lower cone (π) in the 2nd surface Brillouin zone (SBZ) is suppressed by factor of $\sim$50 as compared to its intensity in the 1st SBZ. For the upper cone (π^*) the situation is opposite. Its intensity in the 2nd SBZ is enhanced while in the 1st SBZ it is dramatically suppressed. This scenario is clearly seen in Supplementary Figures 6(a,c) which show zoomed dispersion of Dirac bands along the $\overline{\Gamma} - \overline{\mathrm{K}}$ direction of the SBZ (k passes from 1st to 2nd SBZ through $\overline{\mathrm{K}}$-point). The anisotropy of intensities is so strong that even small angular inaccuracies should cause significant imbalance between intensities of π and π^* peaks.

Since spin resolved data shown in Figure 3 in the article (and in Supplementary Figure 5) was measured in the direction perpendicular to $\overline{\Gamma} - \overline{\mathrm{K}}$ (optimal geometry for elimination of Brillouin zone effects and observation of both sides of Dirac cones), the misalignment

causing unequal intensities of π and π^* in Figure 3(f) corresponds to misalignment along $\overline{\Gamma} - \overline{\mathrm{K}}$. This allows us to estimate the angular misalignment from ARPES data shown in Supplementary Figures 6(a,c).

In Supplementary Figure 6(a) the angular acceptance frame of the spin-ARPES spectrometer used for Figure 3(f) (0.7°) is denoted by a yellow rectangle and positioned precisely at $\overline{\mathrm{K}}$. The EDC profile, representing the corresponding spin-resolved spectrum, is acquired by integration of ARPES intensity within yellow frame and is shown in Supplementary Figure 6(b). In this spectrum peaks of π and π^* bands have equal intensity. In our analysis we have scanned the position of the spectrometer frame along $\overline{\Gamma} - \overline{\mathrm{K}}$ and looked for the intensity variations of π and π^* peaks. The intensity imbalance seen in Figure 3(f) in the article is already achieved for very small misalignment of the spectrometer frame of only 0.12° toward 1st SBZ [Supplementary Figure 6(c,d)]. Hence the error of angular positioning of the sample is truly minor: less than 20% of the acceptance angle of the spin-ARPES spectrometer. (Note that the larger width of the photoemission peaks seen in Figure 3(f) as compared to Supplementary Figure 6 is not due to the angle resolution but due to the energy resolution of the spin-resolved measurements and not relevant for the present analysis.) As an additional argument, we have compared the experimental error with the angular (momentum) localization of the spin hedgehog in the gapped Dirac cone. According to Rakyta et al. (Ref. [18]), the localization region of out-of-plane spins around the $\overline{\mathrm{K}}$-point is given by

$$\Delta k_{\mathrm{S}} = 3\sqrt{2} \times \frac{\lambda_{\mathrm{R}}}{\hbar \nu_{\mathrm{F}}} \tag{1}$$

where ν_{F} is the Fermi velocity of Dirac fermions, and λ_{R} the Rashba parameter for the spin-orbit interaction in the graphene. The Rashba splitting seen in Figures 3(c) and 3(d) in the article (~70-80 meV) means λ_{R}~25-30 meV, which gives for the localization of the hedgehog $\Delta \mathrm{k_S}$~0.015Å^{-1} (or 0.25° at 62 eV photon energy). The angular localization of the spin hedgehog around $\overline{\mathrm{K}}$ is marked in Supplementary Figures 6(a,c) by a red frame. Apparently, the red frame remains perfectly inside of the yellow frame of the spectrometer in the case of 0.12° sample misalignment (and would remain there for even larger errors). This in turn means that the entire spin hedgehog around $\overline{\mathrm{K}}$ is acquired by the spectrometer which confirms the out-of-plane spin obtained in the measurement.

We can also roughly estimate the sensitivity of the spin-resolved measurement and the expected magnitude of out-of-plane spin polarization. Indeed, the angular localization

of the spin hedgehog ($0.25°\times0.25°=0.0625(°)^2$) is much smaller than the overall angular acceptance aperture of the spin-resolved analyser ($0.7°\times0.7°=0.49(°)^2$). Hence the intensity from the hedgehog region is only about $\frac{0.0625}{0.49}$=13% of the overall intensity. This is very well consistent with experimentally measured maximum of the out-of-plane spin polarization which is about 10% [Figure 3(f) in the article].

Supplementary Note 6

Relevance to spintronics

Xiao et al. in Ref. [19] have theoretically predicted a valley Hall effect in graphene with broken inversion symmetry and orbital magnetic moment of opposite sign at $\overline{\mathrm{K}}$ and $\overline{\mathrm{K}'}$ valleys. The effect is illustrated in Supplementary Figure 7(a) for a graphene stripe bearing an electric current. When the electric field $E_{\mathrm{in-plane}}$ is applied in the graphene plane and collinear to the current direction, deflection of the charge carriers toward the edge of the stripe occurs due to valley-polarized scattering induced by a Berry phase effect. This causes different population of Dirac cones at $\overline{\mathrm{K}}$ and $\overline{\mathrm{K}'}$ valleys at opposite edges of the graphene stripe. As a result a transverse voltage V_{VHE} appears. Note that the model of Xiao et al. does not utilize the real spin of charge carriers but the pseudo spin associated with the valley index.

A similar effect was later elaborated theoretically by Tsai et al. [20] but for real spin based on the high-spin-orbit material with broken sublattice symmetry silicene. It was suggested to use an electric field applied perpendicular to graphene in order to create a Zeeman-type splitting of the Dirac cone. Such induced spin-polarization has opposite sign at $\overline{\mathrm{K}}$ and $\overline{\mathrm{K}'}$ valleys. Scattering of electrons to the valleys with spin polarization opposite to the electron spin is suppressed. This spin-valley scattering is expected to be much more effective than the simple valley-only scattering described in Ref. [19] and may allow for nearly 100% filtering of electron spin [20].

The scheme of a possible device utilizing the effect of spin-valley scattering (originally proposed in Ref. [20]) is shown in Supplementary Figure 7(b). This is a spin separator consisting of a Y-shaped flake of graphene attached to three conducting gates/leads. It is assumed that gates 2 and 3 (collectors) have equal potential V_{G2} and V_{G3} , while the

voltage at gate 1 (emitter) V_{G1} is different. The potential difference between V_{G1} and $V_{G2,G3}$ drives electric current through the graphene flake, but, at the same time, creates an in-plane electric field which activates spin-valley scattering. As a result charge carriers with one spin are deflected toward gate 2 (red arrow) and charge carriers with the opposite spin toward gate 3 (blue arrow).

The present case of graphene/Au/Fe(110) is very interesting in the context of such device since it has high spin-orbit splitting and broken sublattice symmetry and out-of-plane spin polarization in the gap of Dirac cones, which according to Ref. [18], changes its sign at $\overline{\mathrm{K}}$ and $\overline{\mathrm{K'}}$ valleys. It is also remarkable that no external electric field is needed to achieve spin polarization of valleys in graphene/Au/Fe(110), since it is induced not by a Zeeman field but through extrinsic spin-orbit interactions.

Although graphene/Au/Fe(110) cannot be directly used for the construction of an effective spin separator since it has conducting substrate and cannot bear 2D currents, it is a useful system allowing to study and understand physics relevant to valleytronic devices and graphene-gate junction therein.

Supplementary Note 7

Preparation of graphene on Fe(110)

All sample preparations were done *in situ*. The Fe(110) substrate was prepared as several tens of monolayers (ML) of Fe grown on W(110). The W(110) crystal was initially cleaned by repeated cycles of annealing in oxygen (partial pressure of oxygen 1×10^{-7} mbar, temperature 1500K) followed by short flashing of the sample up to 2300K in ultra-high vacuum (UHV) environment [1, 2]. The sample preparation is reported in Supplementary Figure 8. Upper panels in Supplementary Figure 8 show band structures of the sample measured at different stages of preparation, while the lower panels display corresponding LEED (low energy electron diffraction) patterns providing structural information. Photoemission intensity in upper panels of Supplementary Figure 8 is enhanced through first or second derivative over energy.

Supplementary Figure 8(a) displays the valence band structure of W(110) measured along the $\overline{\Gamma} - \overline{\mathrm{H}}$ direction of its surface Brillouin zone (SBZ). Cleanliness of the surface was evi-

denced by intense Dirac-cone type [3] dispersion of a surface resonance at 1.5 eV binding energy (at $\overline{\Gamma}$-point) denoted as DC, by the presence of pronounced surface projected band gaps (white dotted lines) as well as by the absence of oxygen-related peaks at 6 eV and carbon-derived bands at 3-5 eV [1]. Alternative indicators of clean W(110) are a surface-induced shift of W 4f core levels [not shown] [1] and a LEED pattern free of any superstructures [lower panel of Supplementary Figure 8(a)].

For the preparation of Fe(110) 20−40 ML of Fe were deposited on clean W(110) at a rate of 0.5ML/min. Right after the deposition Fe shows a very diffuse LEED pattern. In order to reach well-ordered crystalline Fe(110), the sample was annealed at 800K for 5 minutes. Afterwards, a very sharp LEED pattern nearly identical to that of W(110) was observed [lower panel in Supplementary Figure 8(b)] and a pronounced dispersion of Fe 3d bands emerged in the vicinity of the Fermi level [upper panel in Supplementary Figure 8(b)]. The high quality of the obtained Fe(110) is additionally evidenced by the presence of a faint dispersion due to a surface resonance at ∼3 eV (at $\overline{\Gamma}$-point) denoted as SR [17].

Graphene was synthesized by chemical vapour deposition of ethylene or alternatively propylene. The Fe(110) sample was heated to 950-1050K in UHV. Then the hydrocarbon was let into the chamber at a partial pressure of 5×10^{-6} mbar for 10 minutes. The successful synthesis of graphene depends strongly on the partial hydrocarbon pressure and sample temperature. In the case of insufficient control over these parameters an Fe surface carbide is formed. Its valence band structure is shown in Supplementary Figure 8(c). This carbidic phase is characterized by a broad non-dispersing peak at ∼4 eV. The LEED pattern of Fe surface carbide [lower panel in Supplementary Figure 8(c)] reveals a complex constellation of spots and is found to be in agreement with earlier observations by Vinogradov et al. [4]

An ARPES characterization of the successfully synthesized graphene is shown in the upper panel of Supplementary Figure 8(d). The presence of graphene is evidenced by characteristic dispersions of π- and σ-bands emerging at $\overline{\Gamma}$ at 10.1 eV and 4.5 eV, respectively. The LEED pattern of graphene/Fe(110) [lower panel in Supplementary Figure 8(d)] is distinctly different from the carbidic phase and shows a set of 1D-like spot chains determined by the lattice mismatch between graphene and Fe(110). The origin of this moiré pattern is explained in Supplementary Figure 9.

Supplementary Figure 9 plots LEED patterns of asymmetric Fe(110) (blue dashed lines) and graphene (orange dashed lines) extracted from experimental LEED measurements. One

sees that the principle diffraction spots at the corners of LEED patterns (see the area inside of dashed circle) are not in registry with each other due to surface lattice mismatch between Fe and graphene. This structural difference determines the 2D repetition of diffraction spots (black points) with periodicity (7×17) in terms of graphene hexagons [4]. This moiré constellation around a principle graphene spot as it occurs in LEED is zoomed in Supplementary Figure 9(b). The orientational correspondence of LEED to the pseudo-1D stripes of graphene on Fe(110) is emphasized in Supplementary Figure 9(c) where the stripes are sketched (including this longitudinal wave-like pattern). Supplementary Figure 9(a) also plots surface Brillouin zones of Fe(110) and graphene constructed from the LEED patterns. One sees that the $\overline{\mathrm{K}}$-point of graphene is nearly matched to the $\overline{\mathrm{H}}$-point of Fe(110), while the $\overline{\mathrm{K}'}$-point of graphene is very much offset from the $\overline{\mathrm{P}}$-point of Fe. This suggests that electronic hybridization between Fe and graphene must occur differently in the band structure around $\overline{\mathrm{K}}$ and $\overline{\mathrm{K}'}$ points. This was indeed observed by ARPES (Varykhalov et al. Contrasting behavior of Dirac and massive electrons in a graphene superlattice on Fe(110). To be published).

Supplementary Note 8

Synthesis of Au-intercalated graphene on Fe(110)

Intercalation with Au was achieved by deposition of one up to several monolayers of Au on graphene/Fe(110) and subsequent annealing at 750-800K. We have studied two concentrations of Au for which band structures and charge doping of graphene were found clearly defined and homogeneous over the surface. The first phase, showing n-type doping and a 1D electronic structure, is achieved at nominally 1.4ML of intercalated Au. This phase is referred in the manuscript as 1ML-phase. The second phase (charge neutral) is achieved after increasing the total amount of intercalated Au up to 2.3ML. This phase with higher Au concentration is referred in the paper as 2ML-phase. For higher concentrations of intercalated Au we found no differences in the band structure as compared to 2ML.

Supplementary References

[1] Varykhalov, A., Rader, O. & Gudat, W. Structure and quantum-size effects in a surface carbide: W(110)/C-R(15×3). *Phys. Rev. B* **72**, 115440 (2005).

[2] Varykhalov, A., Rader, O. & Gudat, W. Self-organization of one-dimensional Au nanowires on a surface carbide and lateral electron quantization. *Phys. Rev. B* **72**, 241404(R) (2005).

[3] Rybkin, A. G., Krasovskii, E. E., Marchenko, D., Chulkov, E. V., Varykhalov, A., Rader, O. & Shikin, A. M. Topology of spin polarization of the 5d states on W(110) and Al/W(110) surfaces. *Phys. Rev. B* **86**, 035117 (2012).

[4] Vinogradov, N. A., Zakharov, A. A., Kocevski, V., Rusz, J., Simonov, K. A., Eriksson, O., Mikkelsen, A., Lundgren, E., Vinogradov, A. S., Mårtensson, N. & Preobrajenski, A. B. Formation and structure of graphene waves on Fe(110). *Phys. Rev. Lett.* **109**, 026101 (2012).

[5] Marchenko, D., Varykhalov, A., Scholz, M. R., Bihlmayer, G., Rashba, E. I., Rybkin, A., Shikin, A. M. & Rader, O. Giant Rashba splitting in graphene due to hybridization with gold. *Nature Commun.* **3**, 1232 (2012).

[6] Shirley, E. L., Terminello, L. J., Santoni, A. & Himpsel, F. J. Brillouin-zone-selection effects in graphite photoelectron angular distributions. *Phys. Rev. B* **51**, 13614 - 13622 (1995).

[7] Bostwick, A., Ohta, T., McChesney, J. L., Emtsev, K. V., Seyller, Th., Horn, K. & Rotenberg, E. Symmetry breaking in few layer graphene films. *New. J. Phys.* **9**, 385 (2007).

[8] Pletikosić, I., Kralj, M., Pervan, P., Brako, R., Coraux, J., N'Diaye, A. T., Busse, C. & Michely, T. Dirac cones and minigaps for graphene on Ir(111). *Phys. Rev. Lett.* **102**, 056808 (2009).

[9] Sánchez-Barriga, J., Varykhalov, A., Marchenko, D., Scholz, M. R. & Rader, O. Minigap isotropy and broken chirality in graphene with periodic corrugation enhanced by cluster superlattices. *Phys. Rev. B* **85**, 201413 (2012).

[10] Starodub, E., Bostwick, A., Moreschini, L., Nie, S., El Gabaly, F., McCarty, K. F., Rotenberg, E. In-plane orientation effects on the electronic structure, stability, and Raman scattering of monolayer graphene on Ir(111). *Phys. Rev. B* **83**, 125428 (2011).

[11] Marchenko, D., Sánchez-Barriga, J., Scholz, M. R., Rader, O. & Varykhalov, A. Spin splitting of Dirac fermions in aligned and rotated graphene on Ir(111). *Phys. Rev. B* **87**, 115426 (2014)

[12] Rusponi, S., Papagno, M., Moras, P., Vlaic, S., Etzkorn, M., Sheverdyaeva, P. M., Pacilé, D.,

Brune, H. & Carbone, C. Highly anisotropic Dirac cones in epitaxial graphene modulated by an island Superlattice. *Phys. Rev. Lett.* **105**, 246803 (2010).

[13] Zhou, S. Y., Gweon, G. H. & Lanzara, A. Low energy excitations in graphite: the role of dimensionality and lattice defects. *Ann. Phys. (N.Y.)* **321**, 1730 - 1746 (2006) and references therein.

[14] Shikin, A. M., Rybkin, A. G., Marchenko, D., Rybkina, A. A., Scholz, M. R., Rader, O. & Varykhalov, A. Induced spinorbit splitting in graphene: the role of atomic number of the intercalated metal and πd hybridization. *New J. Phys.* **15**, 013016 (2013).

[15] Rashba, E. I. Graphene with structure-induced spin-orbit coupling: spin-polarized states, spin zero modes, and quantum Hall effect. *Phys. Rev. B* **79**, 161409(R) (2009).

[16] Kuemmeth, F. & Rashba, E. I. Giant spin rotation under quasiparticle-photoelectron conversion: joint effect of sublattice interference and spin-orbit coupling. *Phys. Rev. B* **80**, 241409(R) (2009).

[17] Sánchez-Barriga, J., Fink, J., Boni, V., Di Marco, I., Braun, J., Minár, J., Varykhalov, A., Rader, O., Bellini, V., Manghi, F., Ebert, H., Katsnelson, M. I., Lichtenstein, A. I., Eriksson, O., Eberhardt, W. & Dürr, H. A. Strength of correlation effects in the electronic structure of iron. *Phys. Rev. Lett.* **103**, 267203 (2009).

[18] Rakyta, P., Kormányos, A. & Cserti, J. Effect of sublattice asymmetry and spin-orbit interaction on out-of-plane spin polarization of photoelectrons. *Phys. Rev. B* **83**, 155439 (2011).

[19] Xiao, D., Yao, W. & Niu, Q. Valley-contrasting physics in graphene: magnetic moment and topological transport. *Phys. Rev. Lett.* **99**, 236809 (2007).

[20] Tsai, W.-F., Huang, C.-Y., Chang, T.-R., Lin, H., Jeng, H.-T. & Bansil, A. Gated silicene as a tunable source of nearly 100% spin-polarized electrons. *Nat. Commun.* **4**, 1500 (2013), DOI: 10.1038/ncomms2525.
